# Multi-Scale Pixel-Based Image Fusion Using Multivariate Empirical Mode Decomposition

**DOI:** 10.3390/s150510923

**Published:** 2015-05-08

**Authors:** Naveed ur Rehman, Shoaib Ehsan, Syed Muhammad Umer Abdullah, Muhammad Jehanzaib Akhtar, Danilo P. Mandic, Klaus D. McDonald-Maier

**Affiliations:** 1. Department of Electrical Engineering, COMSATS Institute of Information Technology, Park Road, Chak Shahzad, Islamabad 44000, Pakistan; E-Mail: mzaib100@gmail.com; 2. School of Computer Science and Electronic Engineering, University of Essex, Wivenhoe Park, Colchester CO4 3SQ, UK; E-Mails: sehsan@essex.ac.uk (S.E.); kdm@essex.ac.uk (K.D.M.-M.); 3. Halliburton Worldwide Limited, Islamabad 44000, Pakistan; Email: umerabdullah30@ee.ceme.edu.pk; 4. Department of Electrical Engineering, Imperial College London, Exhibition Road, London SW7 2AZ, UK; E-Mail: d.mandic@imperial.ac.uk

**Keywords:** multi-focus image fusion, multi-exposure image fusion, signal decomposition, multivariate empirical mode decomposition, multiresolution analysis, non-subsampled contourlet transform

## Abstract

A novel scheme to perform the fusion of multiple images using the multivariate empirical mode decomposition (MEMD) algorithm is proposed. Standard multi-scale fusion techniques make *a priori* assumptions regarding input data, whereas standard univariate empirical mode decomposition (EMD)-based fusion techniques suffer from inherent mode mixing and mode misalignment issues, characterized respectively by either a single intrinsic mode function (IMF) containing multiple scales or the same indexed IMFs corresponding to multiple input images carrying different frequency information. We show that MEMD overcomes these problems by being fully data adaptive and by aligning common frequency scales from multiple channels, thus enabling their comparison at a pixel level and subsequent fusion at multiple data scales. We then demonstrate the potential of the proposed scheme on a large dataset of real-world multi-exposure and multi-focus images and compare the results against those obtained from standard fusion algorithms, including the principal component analysis (PCA), discrete wavelet transform (DWT) and non-subsampled contourlet transform (NCT). A variety of image fusion quality measures are employed for the objective evaluation of the proposed method. We also report the results of a hypothesis testing approach on our large image dataset to identify statistically-significant performance differences.

## 1. Introduction

Image fusion refers to combining multiple images to produce a single output image that carries the salient features of all fused images. Fusion techniques are particularly relevant in cases where limitations of optical sensors and imaging conditions make it difficult to capture complete detail in a single image [1]. For example, lenses typically have a limited depth of field and cannot focus on two objects placed at different focal lengths [2]. Similarly, for naturally illuminated scenes, the information content in an image depends on the orientation of the light source relative to that of the object of interest; shadows may also cause loss of information [3].

To overcome these impediments, multiple images containing partial information about a scene can be fused to reconstruct its complete information. Image fusion techniques either work in the (I) spatial domain; or in the (II) frequency domain. Spatial domain fusion methods work directly with image (pixels) intensity values. The key steps involved are: (1) the generation of a quantitative map of the information content for each image; (2) comparison of information content at the pixel level; (3) assigning weights to individual pixels (or a set of pixels) based on information content; and (4) weighted recombination to obtain a fused image. An example of the spatial domain fusion method is given in [4], in which the intensity level of the output (fused) pixels are computed from a weighted sum of intensities of corresponding pixels in input images. In [5], input (to-be-fused) images are divided into uniform blocks after which image portions containing the highest contrast are selected in the fused image. Iterative fusion techniques are also popular and obtain an initial estimate of the fused image and then iteratively improve that estimate via spatial domain fusion [6].

Principal component analysis (PCA) offers another avenue to perform spatial domain fusion of multiple images by converting the correlated input images into a set of uncorrelated components. Fusion is achieved by weighted averaging of the components of the images to be fused; the weights for each input image are computed from the eigenvector corresponding to the largest eigenvalue of the covariance matrix of each source image [7]. In [8], pyramid decomposition and the pixel-level fusion were followed by a kernel-based PCA method to obtain the fused image. Similarly, in [9], a higher order singular value decomposition (HOSVD)-based fusion approach was presented. Moreover, neural network-based evolutionary approaches for non-linear parametric identification have been proposed for data fusion [10].

Transform domain fusion techniques, on the other hand, operate by converting input images into their equivalent transform domain coefficients, assigning weights to the coefficients based on information content, selecting the relevant coefficients and finally taking the inverse transform transform [11]. Typical examples are the methods based on Gaussian pyramids [12], fast Fourier transform (FFT) [13], discrete cosine transform (DCT) [14], discrete wavelet transform (DWT) [15], dual tree complex wavelet transform [16], non-subsampled contourlet transform (NCT) [17–19] and shearlet transform [20]. Specifically, in relation to the DWT-based fusion, steerable filters were used in [21] to obtain the wavelet decomposition of the constituent images, and the coefficients carrying the highest energy were selected in the fused image. Morphological operations were performed in [22], in addition to the wavelet transform, to obtain the fused image. In [23], the problem of single image dehazing is addressed via a Laplacian pyramid-based multi-scale approach, while wavelet packets, operating in both the spatial, as well as the transform domain, were used in [24]. In [25], undecimated wavelet transform along with nonorthogonal filter banks were utilised in a multi-scale fusion approach.

While standard transform domain fusion methods have been shown to perform well in many applications, their static filter banks (with fixed cut-off frequencies) and predefined basis functions are the main hurdles in performing the fusion of matched spatial frequency content between multiple input images. These *a priori* assumptions on input images may introduce artefacts in the resulting decompositions, which can affect fusion. Recently, empirical mode decomposition (EMD) has been proposed as a solution to the above problems owing to its data-driven nature [26], as it decomposes input data into a set of intrinsic oscillatory components, known as intrinsic mode functions (IMFs), and in the process, makes no *a priori* assumptions regarding the input data. As a result, EMD has found applications in a variety of areas ranging from image fusion [27] to biomedical engineering [28].

EMD in its original formulation can only handle single channel data, meaning that to be able to use it for image fusion, multiple input images must be decomposed separately [29]. This poses certain problems due to the empirical nature of the univariate EMD, yielding: (1) a different number of extracted IMFs for different input images; and (2) misaligned scales from different input images in the final decomposition. This adversely hinders the fusion process, which requires the sets of IMFs from input images to be matched in terms of their frequency content. To that end, complex/bivariate extensions of EMD have been employed for the fusion of two images [30]. The scheme could be seen as hybrid in that it performs fusion at the level of individual pixels, but on multi-scale components obtained through the bivariate extensions of EMD. However, a fully generic EMD-based algorithm capable of fusing an arbitrary number of images is still lacking. Furthermore, the robustness of the available EMD-based fusion schemes has not thus far been verified on a large database of images and by employing a variety of established spatial and spectral quantitative measures.

In this paper, we present a multi-scale fusion algorithm in order to combine any number of input images using the multivariate empirical mode decomposition (MEMD) algorithm, a generic extension of EMD to multivariate data [31]. MEMD operates directly in multidimensional spaces where the input signal resides, thereby making it a viable choice for the fusion process by avoiding problems, such as mode misalignment and mode mixing encountered in standard EMD. We demonstrate the potential of the proposed scheme by performing fusion and subsequent evaluation on a significantly large database of multi-focus and multi-exposure images. We further compare the results of the proposed method with those obtained from the standard fusion algorithms based on the PCA, DWT and NCT by employing a wide range of image fusion performance measures. Finally, a hypothesis-testing approach is utilised to identify statistically-significant performance differences between different fusion methods.

The paper is organised as follows: Section 2 introduces the empirical mode decomposition algorithm and its multivariate extensions. Section 3 presents the idea and justification of performing image fusion via MEMD. Section 4 presents the proposed algorithm, whereas the experimental results and corresponding analysis are given in Section 5, followed by the conclusions in Section 6. In this paper, we have covered fusion techniques applicable to both multi-focus and multi-exposure images. Classes of specialised algorithms for each application are also available in practise [2,3], but are not discussed here.

## 2. Empirical Mode Decomposition and Its Multivariate Extension

Empirical mode decomposition (EMD) is a data-driven method that decomposes an arbitrary signal *x*(*k*) into a set of multiple oscillatory components, called the intrinsic mode functions (IMFs). The IMFs represent the intrinsic temporal modes (scales) that are present in the input data [26]. The IMFs when added together reproduce the input *x*(*k*), as shown in Equation (1) below; the residual *r*(*k*) does not contain any oscillations and represents a trend within the signal.

(1) $$x\left(k\right)=\sum_{m=1}^{M}c_{m}\left(k\right)+r\left(k\right)$$

The *M* IMFs ${\left\{c_{m}\right\}}_{m=1}^{M}$ are extracted from *x*(*k*) by means of an iterative algorithm known as the sifting algorithm, described in Algorithm 1. The method operates by computing the local mean of *x*(*k*) by taking averages of the upper and lower envelopes of *x*(*k*); these envelopes are obtained by the spline interpolation of extrema (minima and maxima). The local mean, considered as being a slowly oscillating (low frequency) component, is then subtracted from *x*(*k*) to yield a “high-frequency” component. This process is iteratively repeated till the resulting “high-frequency” component obeys one of the stopping criteria of IMF.

The choice of a stopping criterion is important, as over-sifting results in IMFs with uniform amplitude modulations, whereas under-sifted IMFs do not satisfy the mono-component criteria. A typical stopping criterion in EMD stops the sifting after the number of extrema and zero crossings are either zero or differ at most by one for *S* consecutive iterations, where 3 ≤ *S* ≤ 6.



**Algorithm 1** The sifting algorithm for empirical mode decomposition (EMD).
1:Find the locations of all the extrema of *x*(*k*);2:Interpolate (via spline interpolation) between all of the minima (respectively maxima) to obtain the signal envelope passing through the minima, *e_min_*(*k*) (resp. *e_max_*(*k*));3:Calculate the local mean *m*(*k*) = (*e_min_*(*k*) + *e_max_*(*k*))/2;4:Subtract the local mean from the signal to obtain the ‘oscillating’ signal *d*(*k*) = *x*(*k*) − *m*(*k*);5:If the resulting signal *d*(*k*) obeys the stopping criterion, it becomes the first IMF; otherwise, set *x*(*k*) = *d*(*k*) and repeat the process from Step 1 until the first IMF is obtained.


### 2.1. Multivariate EMD

The multivariate empirical mode decomposition (MEMD) algorithm (The free MATLAB code to compute MEMD decompositions, along with some natural multivariate data sets, is available online [32]) extends the standard EMD operation to signals containing multiple input channels [31]. The rationale behind MEMD is to decompose a multivariate signal in terms of high- and low-frequency rotational components in n-dimensional spaces *nD*. Therefore, unlike univariate EMD, which handled oscillations, the emphasis here is on extracting the “rotational components” within input data. The algorithm is useful, since natural multivariate data typically contain rotational modes that require a joint treatment for their meaningful study. Standard univariate EMD algorithms may not yield satisfactory results on such datasets as this approach suffers from: (1) non-uniformity, resulting in a different number of IMFs for different channels; (2) mode mixing, characterised either by an IMF carrying data of different scales or a single scale present in more than one IMF; and (3) mode misalignment, resulting in different modes in the same indexed IMFs from different channels. The above issues pose some major challenges in a more widespread use of EMD in data and image fusion applications [27].

To address the above problems, various extensions of EMD for multivariate data have been proposed, which operate by projecting an input signal in *V* uniformly-spaced directions on a unit *p*-sphere. The corresponding envelopes for each direction are then obtained by interpolating the extrema of the projected signals via component-wise spline interpolation. The resulting envelopes are averaged to obtain the local mean. Similarly to the univariate EMD operation, the local mean is finally subtracted from the input to yield a high-frequency rotational component, and this process is repeated till the resulting component fulfils one of the chosen stopping criteria of an IMF. This way, multiple rotational components (IMFs) are obtained via MEMD.

Specifically, let us consider a sequence of *p*-dimensional vectors **s**(*t*) = {*s*_1_(*t*), *s*_2_(*t*), …, *s_p_*(*t*)}, representing a multivariate signal with *p* components and ${\text{x}}_{\theta_{v}}=\left\{x_{1}^{v},x_{2}^{v},\ldots ,x_{p}^{v}\right\}$ denoting a set of *v* = 1, 2, …, *V* direction vectors along the directions given by angles θ**_v_** = {θ*_v_*_1_, θ*_v_*_2_, … θ*_vp_*_−1_} in ℝ*^p^*, then the multivariate extension of EMD suitable for operating on general non-linear and nonstationary multivariate time series is summarised in Algorithm 2.



**Algorithm 2** Multivariate EMD.
1:Choose a suitable point set for sampling a (*p* − 1)-sphere;2:Calculate a projection, denoted by *q*_θ_*v*__(*t*), of the input signal **s**(*t*) along the direction vector **x**_θ_*v*__, for all *v* (the whole set of direction vectors), giving 
$q_{\theta_{v}}{\left(t\right)}_{v=1}^{V}$ as the set of projections;3:Find the time instants 
${\left\{t_{\theta_{v}}^{i}\right\}}_{v=1}^{V}$ corresponding to the maxima of the set of projected signals 
$q_{\theta_{v}}{\left(t\right)}_{v=1}^{V}$;4:Interpolate [
$t_{\theta_{v}}^{i}$, 
$\text{s}\left(t_{\theta_{v}}^{i}\right)$] to obtain multivariate envelope curves 
${\text{e}}_{\theta_{v}}{\left(t\right)}_{v=1}^{V}$;5:For a set of *V* direction vectors, the mean **m**(*t*) of the envelope curves is calculated as:
(2)$\text{m}\left(t\right)=\frac{1}{V}\sum_{v=1}^{V}{\text{e}}_{\theta_{v}}\left(t\right)$6:Extract ‘detail’ **d**(*t*) using **d**(*t*) = **s**(*t*) − **m**(*t*). If *d*(*t*) fulfils the stoppage criterion [33] for a multivariate IMF, apply the above procedure to **s**(*t*) − **d**(*t*); otherwise, apply it to **d**(*t*).

For **s**(**t**) with *p* > 2 number of channels, the local mean estimate from MEMD, in terms of envelopes *e*{θ_1_,θ_2_,…,θ*_p_*_−1_} in the direction represented by a vector θ = {θ_1_, θ_2_,…, θ*_p_*_−1_} in ℝ*^p^*, is given by:

(3) $$m_{p}\left(t\right)\approx \frac{1}{V_{1}V_{2}\ldots V_{p-1}}\sum_{v_{1}=1}^{V_{1}}\sum_{v_{2}=1}^{V_{2}}\cdots \sum_{v_{p-1}=1}^{v_{p-1}}e\left\{\theta_{v_{1}},\theta_{v_{2}},\ldots ,\theta_{v_{p-1}}\right\}$$

For very large values of *V_p_*, equality holds in the approximation relations given in Equation (3), that is convergence to the ‘true’ local mean is achieved. However, in practical cases, a finite number of direction vectors are employed, suggesting the need to choose those vectors effectively, since the accuracy of the estimated local mean hinges on it. Note that in Equation (3), the product *V*_1_*V*_2_ ⋯ *V_p_*_−1_ represents the total number of projection vectors *V* taken along the *p* − 1-sphere, *i.e.*, *V* = *V*_1_*V*_2_ ⋯ *V_p_*_−1_, where *V_p_*_−1_ denotes the total number of vectors taken along the (*p* − 1)-th axis.

### 2.2. Mode Mixing and Mode Misalignment: EMD vs. MEMD

**Figure 1:**
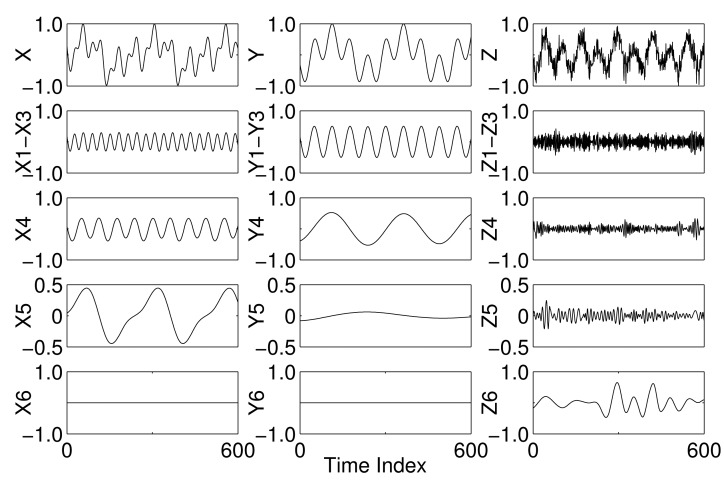
EMD decomposition of a synthetic trivariate dataset showing mode mixing and mode misalignment due to the empirical nature of EMD.

The mode mixing and mode misalignment phenomena in EMD are next demonstrated with the aid of a synthetic trivariate signal composed of a combination of sinusoids, as shown in the top row of Figure 1. A 12-Hz sinusoid was introduced in all components, while a 24-Hz sinusoid was present in the components X and Z and a 4-Hz sinusoid in the components X and Y; the X and Z components were also contaminated with WGN. By applying the EMD algorithm to each component separately, different numbers of IMFs were obtained in each case resulting in mode misalignment, *i.e.*, different scales in the same indexed IMFs of different components. This is evident in all of the IMFs of all of the components, as shown in Figure 1. Mode mixing is also clearly visible in IMF 3 of Channels X and Z and IMF 1 of Channels X and Z. No mode mixing was observed in any of the IMFs of Channel Y, however.

**Figure 2:**
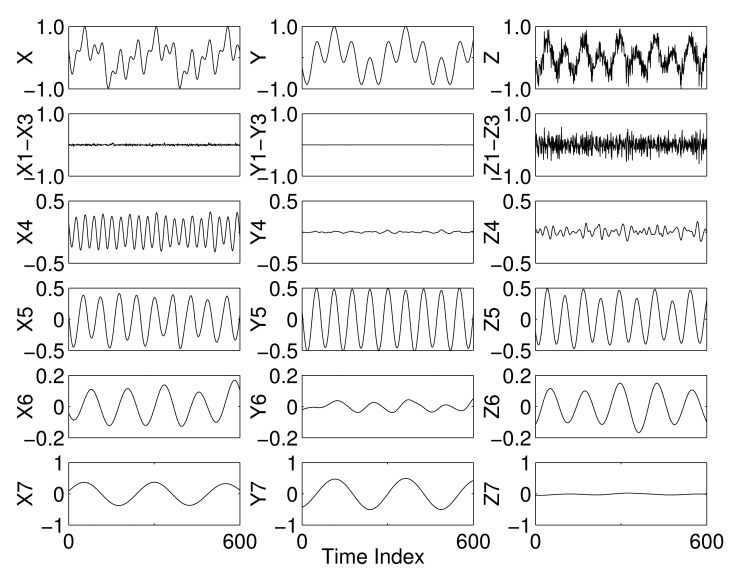
Multivariate EMD (MEMD) decomposition of the same synthetic trivariate dataset as in Figure 1, showing mode alignment and significantly reduced mode mixing.

The MEMD algorithm alleviates the problems of mode mixing and mode misalignment, mainly because of the manner in which the input signals are processed collectively in multidimensional spaces [31]. For illustration, the IMFs obtained by applying MEMD, using *V* = 8 projection vectors, to a multivariate signal containing X, Y and Z as its channels are shown in Figure 2. As evident from the figure, the mode mixing has been largely eliminated within the resulting multivariate IMFs, since each IMF carries a single scale (sinusoid) in all components. In addition, the IMFs from different components are also shown to be aligned in terms to their respective scales: the 12-Hz sinusoid is present in all channels in IMF 5; whereas the 4-Hz and the 24-Hz sinusoids make IMF 7 and IMF 4 in the components X and Y and the components X and Z, respectively.

## 3. MEMD for Image Fusion

In this section, we show the effects of mode mixing and mode misalignment phenomena in EMD-based image fusion process and demonstrate why MEMD is a better candidate for fusion applications. For this purpose, we consider the PAN-sharpening process, which involves the fusion of a panchromatic (PAN) image, providing high-resolution spatial data, but poor spectral resolution, with a multispectral (MS) image, giving poor spatial, but good spectral resolution, to yield an improved MS image exhibiting high spatial and spectral resolutions [34]. In such fusion applications, it is imperative that the multi-resolution components obtained from the PAN and the MS images are aligned in frequency, a feat achieved by MEMD.

**Figure 3:**
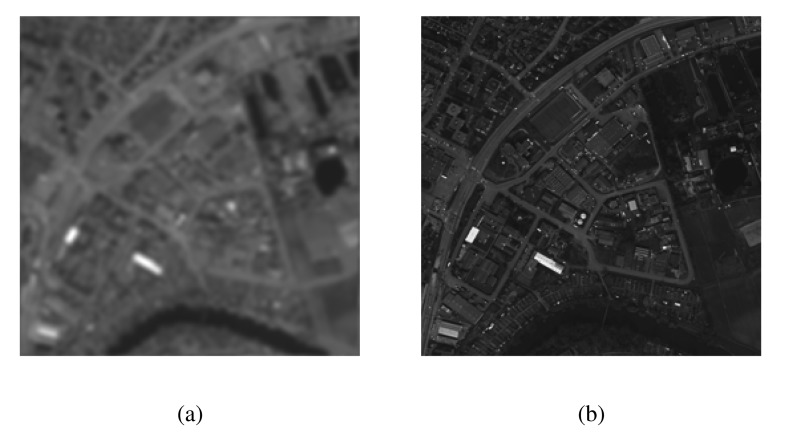
The intensity plane *I* of the low-resolution multispectral (MS) and the high-resolution panchromatic (PAN) images used to demonstrate mode mixing and mode misalignment in EMD. (**a**) The MS image; (**b**) the PAN image.

**Figure 4:**
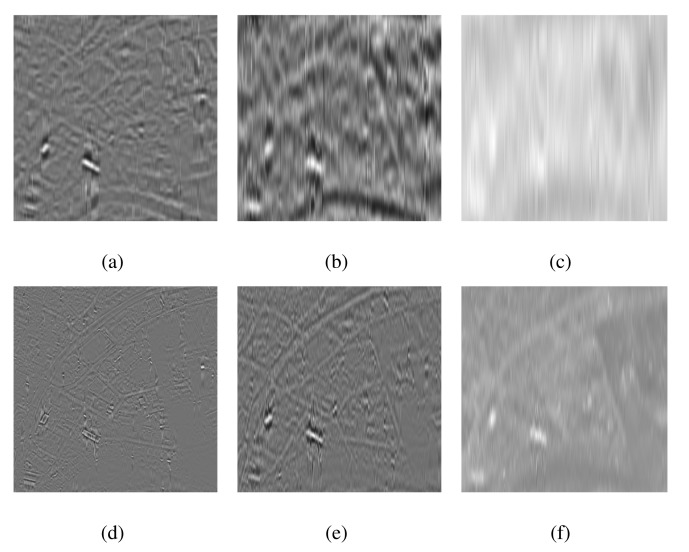
The intrinsic mode functions (IMFs) obtained by applying EMD on the I (top row) and the PAN images (bottom row), shown in Figure 3. (**a**) EMD $IMF_{I}^{1-4}$; (**b**) EMD $IMF_{I}^{5-8}$; (**c**) EMD $IMF_{I}^{9-12}$; (**d**) EMD $IMF_{P}^{1-4}$; (**e**) EMD $IMF_{P}^{5-8}$; (**f**) EMD $IMF_{P}^{9-12}$.

Figure 3a,b respectively shows the intensity *I* plane of the MS and the PAN components of a test image. Both I and PAN images were processed separately in the case of EMD, whereas MEMD was applied to a complex signal, which was created from the intensity component of the MS image vector and the PAN image vector, using *V* = 8 projection vectors. The resulting IMFs (in groups of four) corresponding to the *I* and the PAN images obtained from the EMD algorithm are shown in Figure 4. Observe the artefacts in nearly all sets of IMFs of *I* and PAN, as highlighted in the top row of Figure 4, resulting mainly due to the mode mixing problem. Mode misalignment within the same indexed IMFs from *I* and PAN is also visible, particularly in IMFs 1–4 of *I* ( $IMF_{I}^{1-4}$) and PAN ( $IMF_{P}^{1-4}$), as shown in Figure 4a,d, respectively. It is clear that the $IMF_{P}^{1-4}$ carry high-frequency scales as compared to those in $IMF_{I}^{1-4}$. In the presence of mode misalignment, therefore, it would not make any physical sense to apply a fusion model based on the comparison of spatial content between these sets of IMFs, as it would result in the loss of significant information content from the IMFs containing relatively lower frequencies, that is the information present in $IMF_{I}^{1-4}$ would not be retained in the fused image.

**Figure 5:**
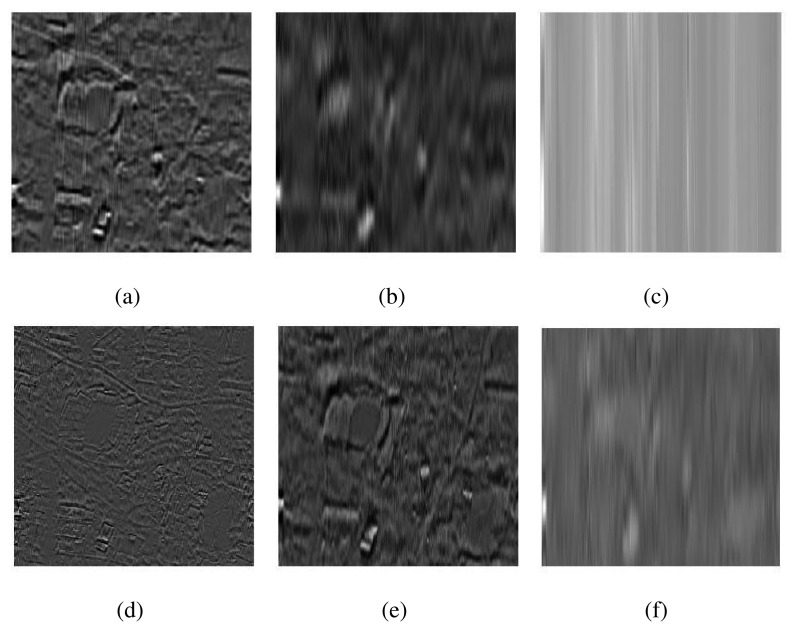
The IMFs obtained by applying MEMD on the *I* (top row) and the PAN images (bottom row), shown in Figure 3. (**a**) MEMD $IMF_{I}^{1-4}$; (**b**) MEMD $IMF_{I}^{5-8}$; (**c**) MEMD $IMF_{I}^{9-12}$; (**d**) MEMD $IMF_{P}^{1-4}$; (**e**) MEMD $IMF_{P}^{5-8}$; (**f**) MEMD $IMF_{P}^{9-12}$.

Figure 5 shows the IMFs (again group of fours) corresponding to the *I* and the PAN images obtained using the MEMD algorithm. Here, as expected, the mode alignment is evident in all pairs of IMFs from *I* and PAN, as the same indexed IMFs carry information related to the same scales. For instance, consider the pair of images in Figure 5a,d corresponding to IMFs 1–4 from I ( $IMF_{I}^{1-4}$) and PAN ( $IMF_{P}^{1-4}$), respectively: the $IMF_{P}^{1-4}$ contains high frequency information, and since, the same frequency contents are not present in the MS image, the $IMF_{I}^{1-4}$ carries very little information. This way, due to the mode alignment, the fusion of *I* and PAN would result in injecting the high-frequency information from the panchromatic image into the fused image, as desired. Similar mode alignment can be observed in the other sets of IMFs, too, facilitating multi-scale fusion at the IMF level using the MEMD algorithm.

## 4. The Proposed Algorithm

The rationale behind the proposed algorithm is to perform fusion at the level of inherent multiple frequency scales of input images. This is accomplished by employing MEMD to decompose all input images into their constituent sub-images based on their frequency contents and performing pixel-based fusion separately at each scale, owing to the mode alignment property of MEMD. Note that the fusion at multiple scales would not make sense in univariate EMD, since it fails to align common frequency scales in input images, as demonstrated in Figures 1 and 4.

The algorithm operates by first converting the *P* input images into a vector form by concatenating their rows/columns. Since MEMD operates on multichannel time-series data, the resulting vectors are then put together to form a multivariate signal containing *P* number of data channels. We next apply MEMD to the resulting signal yielding *M* number of IMFs for each channel; we shall denote the *m*-th IMF of the *p*-th channel (input image) by $I_{m}^{p}\left(a,b\right)$, where *m* = 1 … *M*; *p*= 1 … *P*; and *a* and *b* denote the spatial coordinates of the pixels.

The activity level $A_{m}^{p}\left(a,b\right)$ at each pixel location {*a*, *b*} for the *m*-th IMF of the *p*-th input image is then computed using a window-based approach: that is, a window of size *N* × *N* is centred at the current pixel location {*a, b*}, and some measure of local energy is applied to the pixel values within that window. One popular approach is to estimate the activity level $A_{m}^{p}\left(a,b\right)$ via variance estimate of pixel intensity values by using:

(4) $$A_{m}^{p}\left(a,b\right)={\sum_{i\in I}\sum_{j\in J}\left[I_{m}^{p}\left(a+i,b+j\right)-\mu_{ij}\right]}^{2}$$

where *I* and *J* are sets of horizontal and vertical indices that describe the current window and μ*_ij_* denotes the mean of all of the pixel values inside that window.

Another option is to employ weighted averaging of the absolute values of the pixel intensities within the window by using the following relation:

(5) $$A_{m}^{p}\left(a,b\right)=\sum_{i\in I}\sum_{j\in J}w\left(i,j\right)\times \left|I_{m}^{p}\left(a+i,b+j\right)\right|$$

where *w*(*i*, *j*) is the local weight factor and ∑*_i_*_∈_*_I_* ∑*_j_*_∈_*_J_w*(*i*, *j*) = 1; one could give equal weight to all pixels in an *N* × *N* window resulting in $w\left(i,j\right)=\frac{1}{N^{2}}$, or a kernel function could be used to prioritise the pixels located close to the center point {*a*, *b*}.

Since the activity level *A*(*a*, *b*) quantifies local details in the input images that need to be transferred to the fused image, the sub-images $I_{m}^{p}\left(a,b\right)$ are assigned local weighting factors $WF_{m}^{p}\left(a,b\right)$, which are directly proportional to $A_{m}^{p}\left(a,b\right)$. That is, the IMFs exhibiting greater “activity of interest” locally are assigned higher weights $WF_{m}^{p}\left(a,b\right)$ than those exhibiting lower activity, thereby maximising their contribution to the fused image. We shall call this approach the weighted averaging method. The weight factor ${\left\{WF_{m}^{p}\right\}}_{p=1}^{P}$ corresponding to the *m*-th IMF of the *p*-th channel (input image) is calculated by using the following relation:

(6) $$WF_{m}^{p}\left(a,b\right)=\frac{A_{m}^{p}\left(a,b\right)}{\sum_{p=1}^{P}A_{m}^{p}\left(a,b\right)}$$

In order to obtain the *m*-th IMF of the fused image, the *m*–indexed IMFs of all *P* input images ${\left\{I_{m}^{p}\left(a,b\right)\right\}}_{p=1}^{P}$ are multiplied by their respective weight factors ${\left\{WF_{m}^{p}\left(a,b\right)\right\}}_{p=1}^{P}$ and added together to obtain:

(7) $${\hat{I}}_{m}\left(a,b\right)=\sum_{p=1}^{P}WF_{m}^{p}\left(a,b\right)\times I_{m}^{p}\left(a,b\right)$$

This procedure is repeated for all *M* IMFs to obtain a set of fused IMFs ${\left\{{\hat{I}}_{m}\left(a,b\right)\right\}}_{m=1}^{M}$, which are added together to yield the final fused image *Î*.

(8) $$\hat{I}\left(a,b\right)=\sum_{p=1}^{M}{\hat{I}}_{m}\left(a,b\right)$$

Note that the relations given in Equations (4) and (5) are not the only available options to compute the local activity level in an image using the window-based approach; other popular methods include the rank filter method [15] and the spatial frequency method [35]. Similarly, one could also use other fusion (merging) approaches [15] as an alternative to the weighted averaging scheme given in Equation (6).

**Figure 6:**
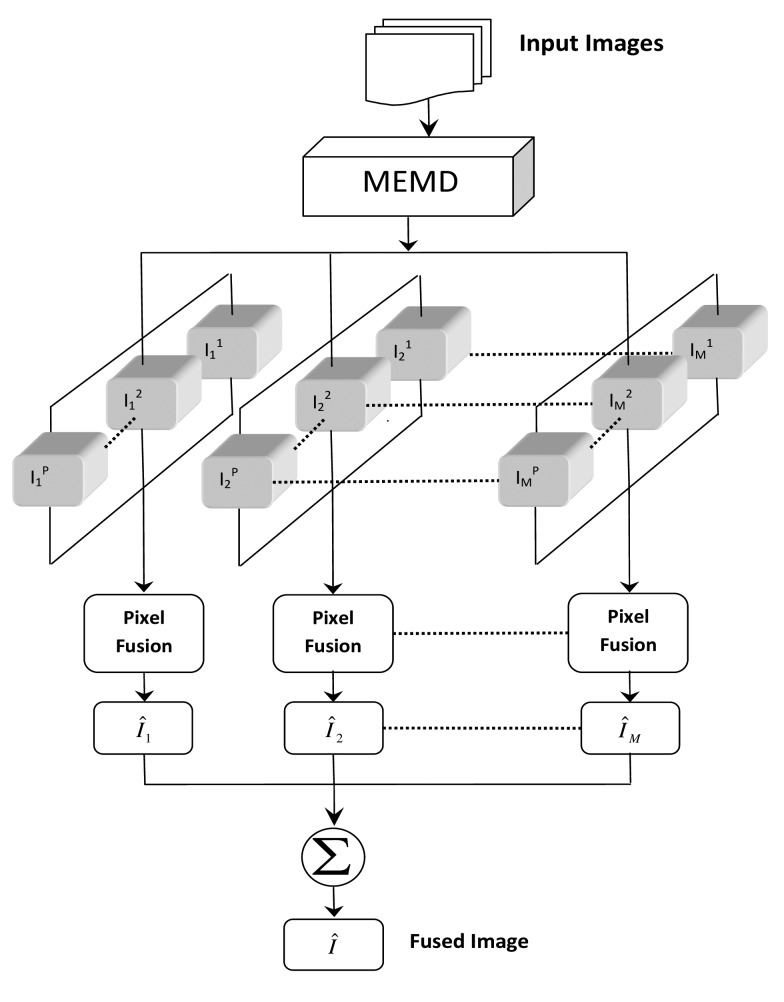
The proposed scheme illustrating the local fusion of *P* arbitrary images, which yields a single fused image *Î*, using the MEMD algorithm.

Figure 6 shows the principle of the proposed method to complement Algorithm 3, which outlines the algorithmic steps involved. Note from Figure 6 that the proposed scheme is inherently multi-scale, with the pixel-based fusion rule/algorithm applied separately for all multi-resolution images (IMFs), yielding *M* fused sub-images ${\left\{{\hat{I}}_{m}\left(a,b\right)\right\}}_{m=1}^{M}$, which are subsequently added to obtain the final fused image.



**Algorithm 3** MEMD-based image fusion algorithm.
1:Convert all input images into a row (or column) vector; stack these vectors to form a multivariate signal, and apply MEMD;2:Reshape the resulting IMFs into its corresponding 2D images, yielding M sub-images 
${\left\{I_{m}^{p}\right\}}_{m=1}^{M}$, for each input image;3:Compute the local activity level 
$A_{m}^{p}\left(a,b\right)$ for each sub-image 
$I_{m}^{p}\left(a,b\right)$ using Equations (4) or (5);4:Calculate local weighting factors 
$WF_{m}^{p}\left(a,b\right)$, based on 
$A_{m}^{p}\left(a,b\right)$, for each 
$I_{m}^{p}\left(a,b\right)$ using Equation (6);5:Obtain the *m*-th fused image *Î_m_* using Equation (7);6:Add all resulting fused images to obtain the final image using Equation (8).


## 5. Results and Analysis

To verify the effectiveness of the proposed fusion algorithm, experiments were performed on a large dataset, for two different fusion paradigms: multi-exposure and multi-focus image fusion. The fusion results obtained from the proposed algorithm were compared against the standard and state-of-the-art in image fusion, namely the principal component analysis (PCA), discrete wavelet transform (DWT) and non-subsampled contourlet transform (NCT) using both single direction (NCT-S), which is the same as the critically-sampled DWT, and multiple directions (NCT-N). For rigour, both qualitative and quantitative evaluation results have been reported. Using both variants of NCT would help us to assess the advantage of using decompositions along multiple directions in the fusion process.

We captured images of 60 different scenes to build a database of multi-focus images; seven images were taken of each scene with different parts of the scene kept out of focus in each image. The aim was to fuse these multiple images to obtain a single in-focus image.

The multi-exposure image dataset used in this work was the robot data set recorded at the Technical University of Denmark [36]. It consisted of 60 image sets with each set consisting of a further 119 images captured from different angles (positions); 19 white LEDs were used in the experiments for illumination/exposure. We selected seven such positions for each scene resulting in the same number of input multi-exposure images for each of the 60 scenes; the positions were chosen such that the partial scene information was obscured/hidden at different locations of the image scene, either due to the over-exposure or under-exposure to light. The reference (ground truth) image for each scene was also available in this case. Since the ground truth corresponds to 119 input exposure images and we had used only a subset (seven) of those input images, the fused image and the ground truth exhibited comparatively low correlation scores. However, since this trend was uniformly observed in all of the techniques used in this work, we could use the ground truth for quantitative evaluation purposes, thus, allowing for a better quantitative evaluation of multi-exposure fusion results.

All simulations were implemented in MATLAB 7.12 and run on an Intel(R) 2.33 GHz machine with 4 GB of RAM. Unless otherwise specified, the parameters used in the MEMD algorithm were: the number of direction vectors *V* = 64; the stopping criteria parameters [33] were [θ_1_ = 0.075, θ_2_ = 0.75, *α* = 0.075] with *M* = 5 number of IMFs. Note that while the number of direction vectors and stopping criterion parameters used in our experiments are standard values widely employed in EMD experiments, the choice of using *M* = 5 IMFs was mainly motivated to enable a fair comparison with the DWT- and NCT-based fusion methods, since they employed five decomposition levels. For the DWT fusion method, Daubechies' wavelet *dB*4 was used with five decomposition levels. For the NCT-based fusion schemes, diamond max-flat filters were used as directional filters, and the filters derived from maximally flat mapping function with four vanishing moments were used as 2D pyramid filters [17]. Moreover, five decomposition levels were used in both cases, with the additional 2, 4, 8, 16 and 32 directions used in the NCT-N from coarser to finer scale. Note that using a greater number of directions within NCT results in an overall much greater number of decompositions as compared to the MEMD, which only gives decomposition in a single direction. Therefore, it would be more reasonable to compare the results of MEMD against NCT-S, as both do not utilise directional decompositions. In MEMD-, NCT- and DWT-based fusion schemes, the local activity level was computed using Equations (4) and (5) for multi-focus and multi-exposure fusion, respectively. This choice was motivated by the analysis of the fusion results obtained from both cases (Equations (4) and (5)), for all multi-scale methods used in our experiments: Equation (4) yielded better results consistently for multi-focus fusion, whereas Equation (5) produced improved multi-exposure results for all multi-scale methods. The square window length of *N* = 9 was chosen in all experiments involving all three methods. The details of the PCA-based fusion algorithm implemented in our case can be found in [7].

### 5.1. Qualitative Evaluation

We now present a qualitative evaluation of different fusion schemes for both multi-exposure and multi-focus image fusion cases:

#### 5.1.1. Multi-Exposure Fusion: Qualitative Analysis

**Figure 7:**
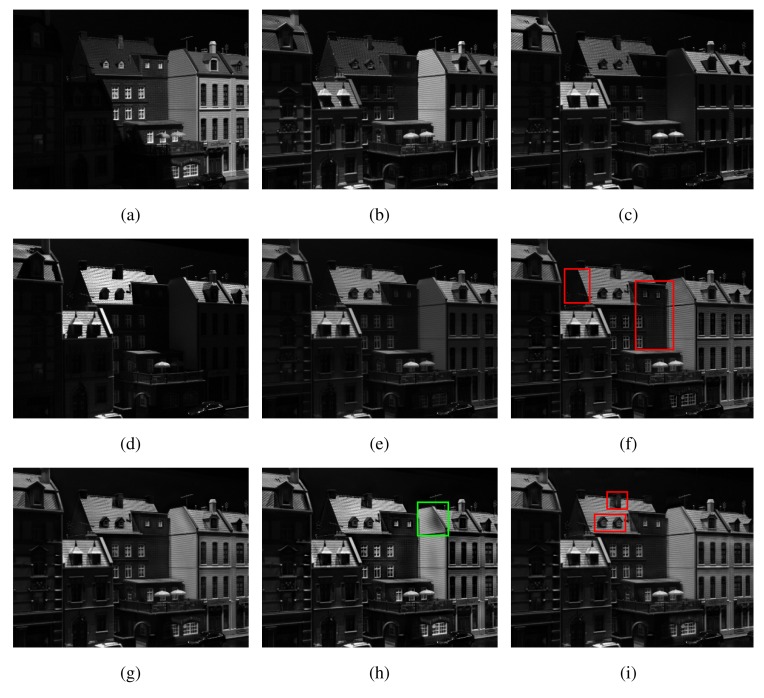
Multi-exposure image fusion results for Dataset 1. (**a**) Image 1; (**b**) Image 2; (**c**) Image 3; (**d**) Image 4; (**e**) PCA; (**f**) DWT; (**g**) non-subsampled contourlet transform using a single direction (NCT-S); (**h**) NCT-N; (**i**) MEMD.

Figure 7a–e shows a subset of one of the datasets utilised in the DTU robot dataset-based experiment. This is an example of a multi-exposure dataset where, within each of the seven input images, different locations of the scene were illuminated by a light source; only four of the total seven input images are shown here due to space restrictions. The input images were fused using the PCA, DWT, NCT-S, NCT-N and the proposed MEMD algorithm, yielding the fused images, which are respectively shown in Figure 7e–i. Please notice that the output images from both variants of NCT (Figure 7g,h) and MEMD (Figure 7i) mostly capture the scene information well, though the MEMD-based fused image appears to be better illuminated and focussed especially in the areas that are highlighted by the rectangular boxes shown in the Figure 7i. Furthermore, note that the fused image obtained from NCT-N has few visible colour artefacts as highlighted by the rectangular box in Figure 7h. The PCA-based fused image shown in Figure 7e is poorly illuminated, whereas the DWT-based fused image (Figure 7f), though much better than the PCA, does not compare well against either the NCT- or the MEMD-based fused images, in terms of overall luminosity (exposure) of the fused image; this is particularly evident in areas highlighted by the rectangular boxes shown in Figure 7f.

Figure 10a–e shows a subset of another input dataset, which has been used in our simulation study. The corresponding fused images from the PCA, DWT, NCT-S, NCT-N and MEMD are shown in Figure 10e–i, respectively. The pattern observed in the dataset of Figure 7 is also present here, that is the NCT- and the MEMD-based fused images appear to be better illuminated as compared to their PCA and DWT counterparts. The NCT-N fused image shows few distortions, as shown in the rectangular box in Figure 10h, though it appears to produce a slightly more sharp image (especially the text) as compared to all other methods. Once again, the visual analysis of the fused image reveals that the PCA shows the worst performance among all fusion methods; DWT shows better performance than PCA, but again falls short of the NCT and MEMD methods. It is hard to detect a significant difference between the fused images from the NCT- and MEMD-based algorithms, though the fused image from MEMD (shown in Figure 10i) appears to be better illuminated along the area highlighted by the rectangular box.

#### 5.1.2. Multi-Focus Fusion: Qualitative Analysis

**Figure 9:**
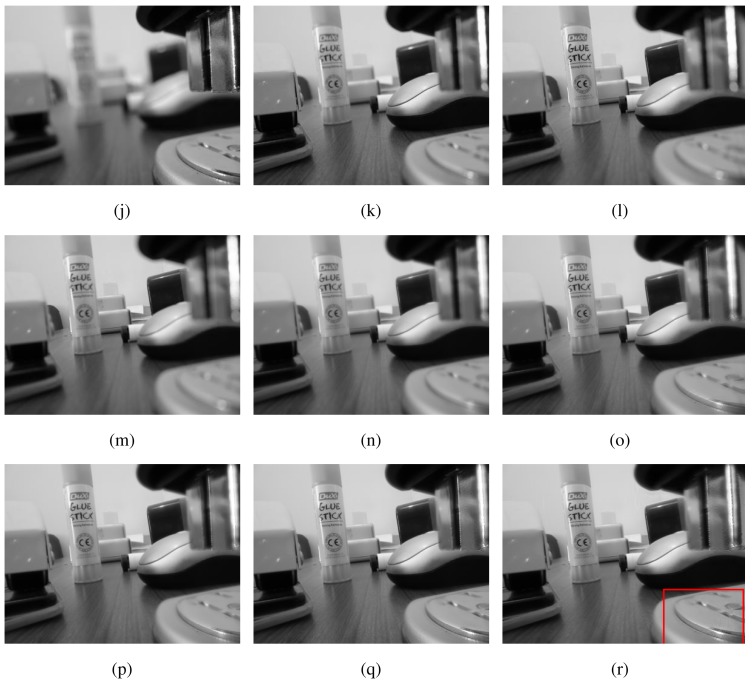
Multi-focus image fusion results for Dataset 1. (**a**) Image 1; (**b**) Image 2; (**c**) Image 3; (**d**) Image 4; (**e**) PCA; (**f**) DWT; (**g**) NCT-S; (**h**) NCT-N; (**i**) MEMD.

Figure 9a–d shows images from one of the 60 multi-focus datasets used in this work. Observe that all input images have different areas within depths of visual focus. The fused images from different methods are shown in Figure 9e (PCA), Figure 9f (DWT), Figure 9g (NCT-S), Figure 9h (NCT-N) and Figure 9i (MEMD). A visual analysis of the fused images highlights the superiority of the MEMD-based fusion method, especially in the region (near the camera), which is highlighted by the rectangular box in the Figure 9i. The MEMD-based fused image is better focussed in that area when compared with all other fused images. Note that the PCA performed worst in that region followed by the DWT. For medium to distant objects, all methods performed well.

**Figure 10:**
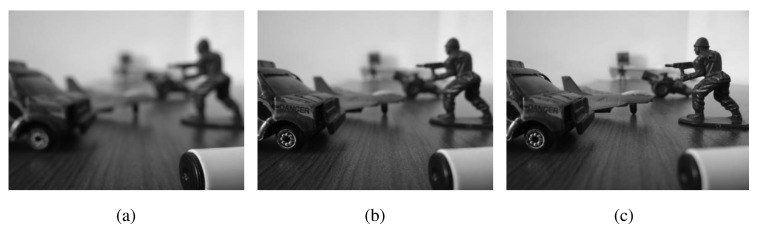

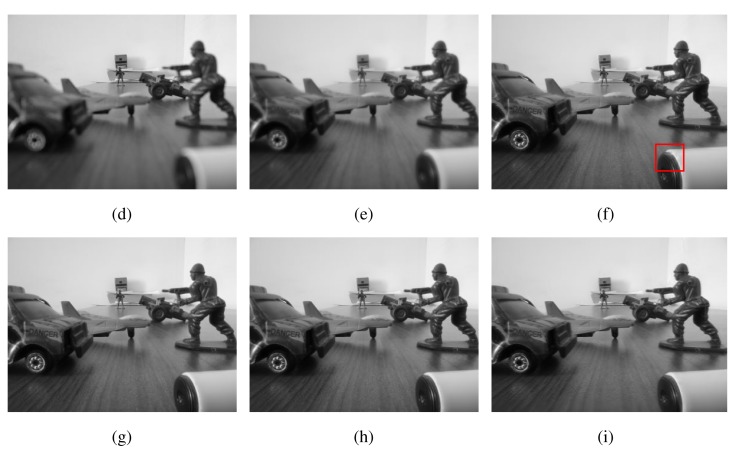
Multi-focus image fusion results for Dataset 2. (**a**) Image 1; (**b**) Image 2; (**c**) Image 3; (**d**) Image 4; (**e**) PCA; (**f**) DWT; (**g**) NCT-S; (**h**) NCT-N; (**i**) MEMD.

Figure 10a–d shows input images in an indoor setting where different objects lying on a table appear in focus within different input images. Due to space restrictions, only five of the total seven input images are shown. The fused images from the five schemes, the PCA, DWT, NCT-S, NCT-N and MEMD, are shown respectively in Figure 10e–i. It can be seen that the DWT-, NCT- and MEMD-based methods produced sharper and overall perceptually better looking images than the PCA method, which failed to focus the near objects. However, some artefact were observed in the DWT-based fused image in the region highlighted by the rectangular box in Figure 10f: notice the spreading of white pixels in the highlighted area. The NCT- and MEMD-based methods produced excellent quality images, while the NCT appears to keep the near objects slightly in better focus as compared to the MEMD.

### 5.2. Quantitative Evaluation

For an objective comparison of the fusion results obtained from the different schemes, we used entropy (*E*), spatial frequency (*SF*) [37], the universal image quality index (*UIQI*) [38], the Piella metric (*PM*) [39] and objective image fusion ( $Q_{p}^{AB/F}$) [40] in our experiments.

The large set of images and a wide range of quality metrics used in our work also enabled us to perform statistical tests to determine whether the differences between the results from various fusion methods were statistically significant or not. For that purpose, we used McNemar's statistical test on a 2×2 classification table for testing the difference between two methods when applied over the same sets of data [41]. The classification table, as shown in Table 1, tabulates the outcome of two methods on a sample of *n* datasets.

In Table 1, *a′* and *d′* denote respectively the number of cases (out of total *n*) where both Method 1 and Method 2 fail and pass; *b′* and *c*′ denote the discordant cases when either of the two methods fails. Note that the total number of test datasets *n* = *a′* + *b′* + *c′* + *d′*.

McNemar's test is based on a null hypothesis that the two marginal probabilities for each outcome are the same (*i.e.*, ℍ_0_ : *p_b′_* = *p_c′_* ), implying that no significant difference exists between the outcome of the two methods. McNemar's test statistic is $\chi^{2}=\frac{{\left(b\prime -c\prime \right)}^{2}}{b\prime +c\prime }$, which has a chi-squared distribution for sufficient discordant pairs *b′* + *c′* > 25. For *b′* + *c′* < 25, a cumulative binomial distribution is used instead of the chi-squared distribution. If the resulting *p*-value is less than the conventional 0.05, the conclusion can be made that there is a significant difference between the two methods.

To implement the McNemar's test, one needs a criterion to determine whether the application of a method on a given dataset resulted in a success (pass) or a failure (fail). Naturally, this criterion should be based on a threshold vector τ determined by suitable values of the quantitative fusion quality measures. In our experiments, we used thresholds on *E*, $Q_{p}^{AB/F}$ and *UIQI/PM* to determine τ. The threshold vectors for multi-exposure and multi-focus fusion were selected to be $\tau_{\text{e}}=\left[\begin{array}{ccc} E>4.55, & \text{UIQI}>0.55, & Q_{p}^{AB/F}>0.45 \end{array}\right]$ and $\tau_{\text{f}}=\left[\begin{array}{ccc} E>7.20, & PM>0.75, & Q_{p}^{AB/F} \end{array}>0.55\right]$, respectively. The threshold values were chosen after thorough visual analysis of the fused images obtained from applying all four methods on a large image set and careful observation of corresponding values of quantitative metrics. For instance, we found that for multi-exposure datasets, the *UIQI* score must be greater than 0.55 (*i.e.*, *UIQI* > 0.55) for the fused image to exhibit little or no distortion and encompass maximum information from input images. Other values of the thresholds were chosen based on the similar observations of different performance measures and the corresponding fused images. For a method to be ‘successful’ on a dataset, all entries of its corresponding τ vector should be equal to unity.

Note that we have not based our quantitative evaluation on the percentage increase in values of the quantitative measures (*UIQI*, *PM*, *etc.*) for different fusion methods, since even a very small change in their values can have a significant effect on the quality of the fused image. Owing to this fact, our evaluation is based on the number of input datasets for which our method outperforms other comparative fusion methods, based on the threshold vectors τ.

#### 5.2.1. Multi-Exposure Fusion: Quantitative Analysis

For quantitative evaluation of multi-exposure fusion results, we implemented the PCA-, DWT-, NCT-S-, NCT-N- and MEMD-based fusion schemes on 60 data scenes from the DTU Robot dataset [36], with seven input multi-exposure images in each set. We had observed from the qualitative analysis of multi-exposure images that the PCA- and the DWT-based schemes failed to produce suitable fused images in terms of luminosity and proper exposure. In comparison, the NCT- and the MEMD-based methods yielded images that were better illuminated, with MEMD outperforming NCT-S slightly in terms of overall exposure and sharpness of the final image. These observations are next confirmed via quantitative quality measures.

**Figure 11:**
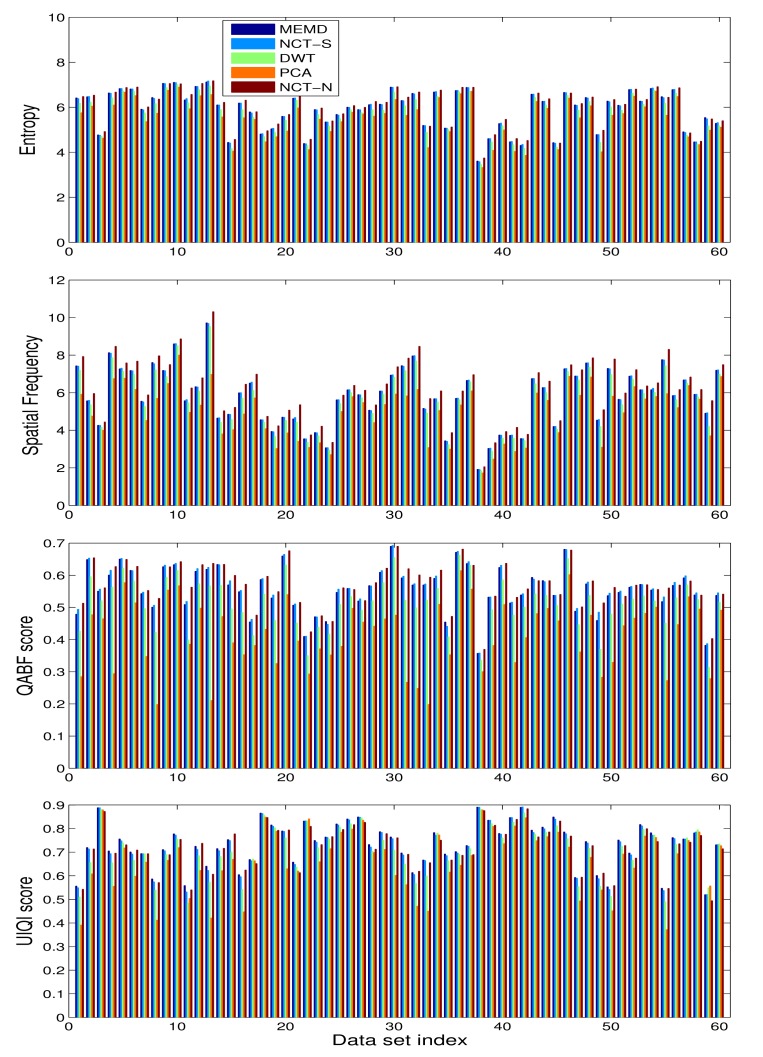
Quantitative analysis on multi-exposure images: bar graphs of the values of quantitative measures, including the *E*, *SF*, $Q_{p}^{AB/F}$ and *UIQI*, shown respectively from the top to the bottom, are obtained for MEMD-, NCT-S-, DWT-, PCA- and NCT-N-based fusion methods.

Figure 11 presents bar graphs showing the scores of *E*, *SF*, $Q_{p}^{AB/F}$ and *UIQI* performance measures for PCA-, DWT-, NCT-S-, NCT-N- and MEMD-based fused images, obtained for the 60 input multi-exposure datasets used in our experiments. It is evident from the figure that the NCT and the proposed MEMD method performed significantly better than the PCA and the DWT schemes for all given performance metrics and datasets. Between the NCT-S- and MEMD-based methods, the MEMD yielded superior performance for the *E*, *SF* and (especially) *UIQI* measures, whereas the NCT-S performed slightly better for the $Q_{p}^{AB/F}$ measure. The performance of the NCT-N method was found to be better for all quantitative measures, except for UIQI, which was expected, since NCT-N employed multiple direction levels along its decompositions. The quantitative analysis confirms the observations made during the visual analysis of Figures 7 and 10.

We next present the results of McNemar's statistical test for multi-exposure datasets in Table 2, in order to verify whether the difference between the results from the proposed method and the other standard methods was statistically significant or not. For that purpose, the performance of the MEMD method was judged separately against the PCA, DWT, NCT-S and NCT-N methods. From Table 2, the MEMD produced superior results as compared to the PCA and the DWT, as the corresponding *p*-values for both cases were less than the threshold of 0.05, thus rejecting the null hypothesis (of no significant difference between the two methods). Furthermore, notice that out of the 60 datasets, there were respectively 18 and 10 discordant datasets where MEMD “passed” (+) and PCA and DWT “failed” (−), whereas there was no dataset where MEMD “failed” (−) and PCA (or DWT) “passed” (+). In terms of percentage, therefore, MEMD showed improved results over PCA and DWT for 30% and 16% of the datasets, respectively. Between NCT-S and MEMD, the null hypothesis of no significant difference between the methods could not be rejected, though MEMD was overall better with five datasets for which MEMD “passed” (+) and NCT-S “failed” (−), and none for which MEMD “failed” (−) and NCT “passed” (+). The comparison between NCT-N and MEMD was more even with two discordant datasets where MEMD “passed” (+) and NCT-N “failed” (-) and *vice versa*. The *p*-value of unity also indicated that the two methods were on par, which was expected given the visual and quantitative analysis of these methods for multi-exposure data.

#### 5.2.2. Multi-Focus Fusion: Quantitative Analysis

The quantitative evaluation of the proposed scheme against PCA, DWT, NCT-S and NCT-N was performed on 60 multi-focus image sets, with each set containing seven input images. We employed *E*, *SF*, $Q_{p}^{AB/F}$ and *PM* metrics for this purpose. Note that instead of the *UIQI* metric, we used its variant *PM* for multi-focus image sets, due to the non-availability of the reference (ground truth) images for these sets.

**Figure 12:**
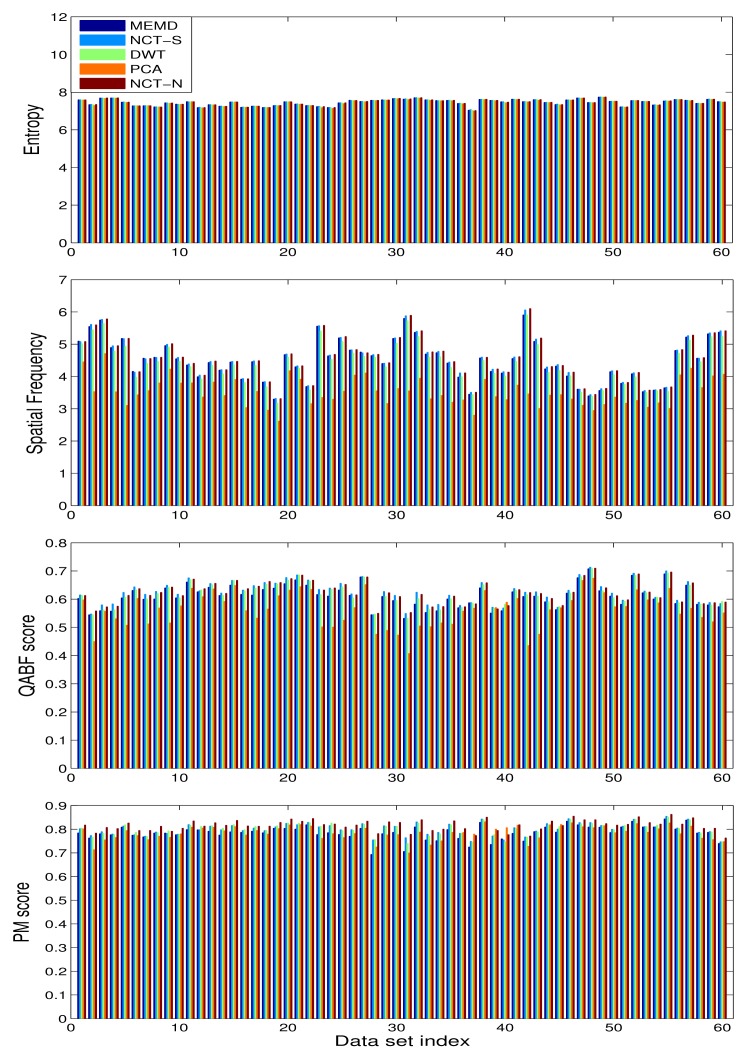
Quantitative analysis on multi-focus images: bar graphs of the values of quantitative measures, including the *E*, *SF*, $Q_{p}^{AB/F}$ and *PM*, shown respectively from the top to the bottom, are obtained for MEMD-, NCT-S-, DWT-, PCA- and NCT-N-based fusion methods.

Figure 12 presents bar graphs showing the metric values for each of the 60 multi-focus datasets, for the *E*, *SF*, $Q_{p}^{AB/F}$ and *PM* measures, shown from top to down, respectively. All of the methods reported *E* values in a close range, whereas for the *SF* measure, the NCT- and the MEMD-based methods showed markedly better results than the DWT- and the PCA-based schemes. For the $Q_{p}^{AB/F}$ measure, the NCT-based methods performed better than all of the other methods, including the MEMD. For the *PM* metric, the NCT-N exhibited comparatively better results.

The results of McNemar's test shown in Table 3 further validate the observations made during the visual and quantitative analysis of multi-focus image sets. As expected, results from the MEMD-based scheme proved to be significantly (and statistically) better than those obtained from the PCA: in total, there were 17 datasets for which MEMD passed and PCA failed, yielding a negligibly small *p*-value. No statistically significant difference was found between the results of MEMD and DWT, NCT-S and NCT-N. By observing the *p*-values in Table 3, however, it can be noticed that the DWT was on par with MEMD, with the *p*-value = 1. Both of the NCT-based variants (employing a single and multiple directions) gave slightly better results than the MEMD, but the improvement offered by NCT over MEMD was not found to be statistically significant, since in both cases, the *p*-value was found to be greater than 0.05.

## 6. Conclusions and Discussion

We have introduced a method to perform the fusion of multiple images using the multivariate empirical mode decomposition (MEMD) algorithm. The proposed method can be viewed as both multi-scale and local (pixel-based); it is fully data adaptive and is also able to simultaneously process any number of input images. The method operates by first employing MEMD to decompose input images into their constituent scale images (multi-scale approach) and subsequently applying a pixel-based fusion scheme to the resulting sub-images, obtaining a fused image containing all relevant details from the input images. We have also shown that the proposed method overcomes the mode mixing and mode misalignment issues observed in standard univariate EMD algorithms and is, hence, better suited for data fusion applications than the standard EMD.

Here, the desirable properties of MEMD have been exploited for multi-scale image fusion, including the multi-exposure and multi-focus image fusion. A qualitative and quantitative evaluation of the proposed method has been performed on a large set of multi-exposure and multi-focus images, and comparisons with the standard fusion methods, such as the PCA, DWT and NCT, have been made using a variety of both spectral and spatial quantitative measures. The large image data base and the range of quantitative measure have enabled us to also perform McNemar's statistical test to verify whether the results from the proposed method are statistically better than those obtained from the standard fusion approaches. We have reported significantly better performance of our method against the PCA for both multi-exposure and multi-focus cases and against the DWT for multi-exposure image sets. When compared against NCT, which is the state-of-the-art in image fusion, the proposed method showed no statistically-significant difference, therefore clearly confirming its potential.

One potential area of improvement in the proposed fusion method is its computational complexity. The proposed method is based on (M)EMD computation, which is inherently computationally more expensive [42] than the PCA, DWT and NCT methods. However, future improvements in (M)EMD computing time will bring down the computational cost of the proposed fusion method; in that context, a number of algorithmic improvements [28] and implementations have already started to emerge. Another improvement could include a pre-processing step of image denoising before application of the proposed method.

## Figures and Tables

**Figure 8:**
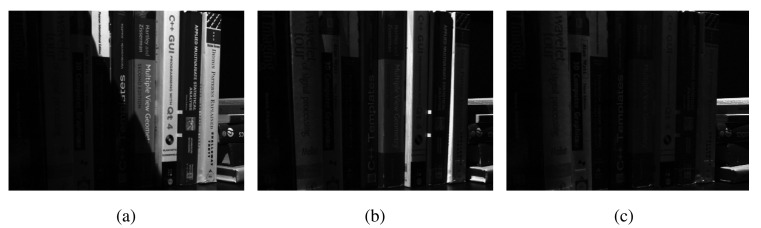

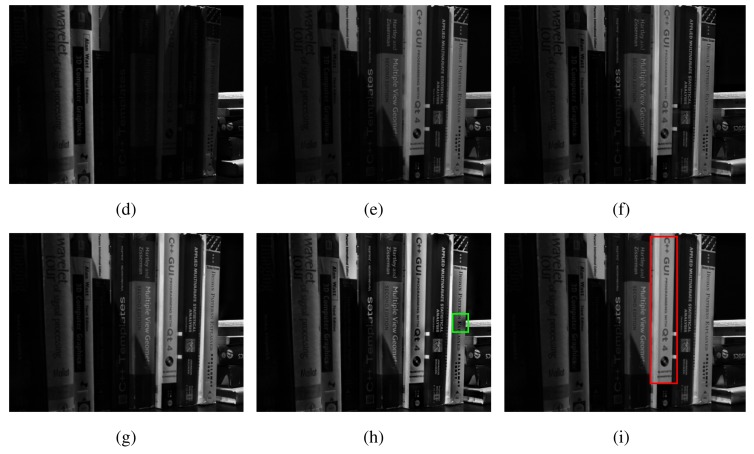
Multi-exposure image fusion results for Dataset 2. (**a**) Image 1; (**b**) Image 2; (**c**) Image 3; (**d**) Image 4; (**e**) PCA; (**f**) DWT; (**g**) NCT-S; (**h**) NCT-N; (**i**) MEMD.

**Table 1:** McNemar's 2 × 2 classification table.

	**Method 2 Fail**	**Method 2 Pass**
Method 1 Fail	*a′*	*b′*
Method 1 Pass	*c′*	*d′*

**Table 2:** McNemar's statistical test results for multi-exposure data.

	**PCA−**		**PCA+**	**DWT−**		**DWT+**	**NCT-S−**		**NCT-S+**	**NCT-N−**		**NCT-N+**
MEMD (−)	9		0	9		0	9		0	7		2
MEMD (+)	18		33	12		39	5		46	2		49
*p* Value		< 10^−4^			**0.0005**			**0.062**			**1.0**	

**Table 3:** McNemar's statistical test results for multi-focus data.

	PCA−		PCA+	DWT−		DWT+	NCT-S−		NCT-S+	NCT-N−		NCT-N+
MEMD (−)	9		0	8		1	6		3	4		5
MEMD (+)	17		34	1		50	0		51	0		51
*p* Value		< 10^−4^			**1.0**			**0.25**			**0.0625**	
